# Relationship between Occupational Metal Exposure and Hypertension Risk Based on Conditional Logistic Regression Analysis

**DOI:** 10.3390/metabo12121259

**Published:** 2022-12-14

**Authors:** Huiling Qian, Guangming Li, Yongbin Luo, Xiaolei Fu, Siyu Wan, Xiaoli Mao, Wenjun Yin, Zhiteng Min, Jinfeng Jiang, Guilin Yi, Xiaodong Tan

**Affiliations:** 1 Department of Preventive Medicine, School of Public Health, Wuhan University, Wuhan 430072, China; 2 Wuhan Prevention and Treatment Center for Occupational Diseases, Wuhan 430015, China; 3 School of Health and Nurse, Wuchang University of Technology, Wuhan 430063, China

**Keywords:** metals, hypertension, occupational exposure, risk

## Abstract

Occupational exposure is a significant source of metal contact; previous studies have been limited regarding the effect of occupational metal exposure on the development of hypertension. This study was conducted to assess the levels of exposure of certain metals (chromium (Cr), iron (Fe), manganese (Mn), and nickel (Ni)) in hypertensive and non-hypertensive workers and to assess the relationship between the risk of hypertension and metal exposure level. Our study included 138 hypertensive patients as case groups and 138 non-hypertensive participants as controls. The exposure risk level was divided according to the limit value after collecting and testing the metal dust in the workshop. Considering the influence of single- and poly-metal, single factor analysis and conditional logistic regression analysis of poly-metal were carried out. The results of the model indicated that the incidence of hypertension increased with an increase in Cr exposure level, and the risk of hypertension was 1.85 times higher in the highest exposure than in the lowest exposure (95% CI: 1.20–2.86, *p* < 0.05). Mn has the same effect as Cr. There was no significant correlation between Fe or Ni and hypertension. Our findings suggested that Cr and Mn exposure in the work environment might increase the risk of hypertension, while no effect of Fe and Ni on blood pressure was found. Prospective study designs in larger populations are needed to confirm our findings.

## 1. Introduction

The number of people with hypertension has doubled since 1990, and 1.28 billion adults worldwide have hypertension currently [1]. In addition to increasing age, risk factors for hypertension also include unhealthy diets, lack of physical exercise, obesity, alcohol consumption, etc. [2]. Epidemiological data suggested a potential relationship between environmental metal exposure and hypertension [3]. Currently, occupational exposure is an important route of metal exposure.

Occupational metal exposure mainly comes from smelters and welders, and metal dust in the working environment damages the health of these workers. For example, welding fumes containing cadmium, Fe, lead, and zinc could affect respiratory health [4], exposure to metal fumes was linked to deterioration of lung function, and occupational Mn exposure might impair immune function, etc. [5,6]. Metal exposure not only causes the above health damage, but also has a certain impact on cardiovascular function. Some studies have found that serum concentrations of copper, selenium, and Cr may be independently associated with hypertension, as well as a high serum Cu–Zn/Mg–Mn ratio [7]. Long-term residence in areas with high exposure to arsenic, Cr, and Mn has a higher risk of hypertension, and exposure to selenium is a protective factor for hypertension [8]. Exposure to magnesium, Fe, and calcium may increase the risk of hypertension or raise blood pressure levels [9]. The increase in diastolic blood pressure levels may be related to mercury exposure [10]. A review discussed the mechanisms of hypertension, suggesting that cadmium and mercury may play a key role in the development of hypertension by inducing endothelial dysfunction [11]. Several studies have also shown a progressive dose–response relationship between lead-exposed workers and blood pressure [12] and that low-level lead exposure increased blood pressure and might increase the risk of hypertension [13]. Other studies have shown that Mn in urine could control blood pressure and prevent hypertension, and in a national study of older Americans, Mn levels were negatively correlated with cardiovascular disease risk [14]. A study of cardiovascular risk in patients with diabetes showed that lower plasma Cr was significantly inversely associated with blood pressure [15].

There are various methods for the detection of heavy metal ions, and the traditional method was used in this study. Although this method could quantify heavy metal ions with high sensitivity and accuracy, it requires professionals to conduct on-site and routine environmental monitoring, and the complex sample pretreatment and expensive equipment limit its application [16]. In addition, the monitoring of airborne metals depends on the stability of the testing instruments [17]. More importantly, air monitoring is only one aspect of exposure and cannot cover all sources of exposure, such as skin, food, soil, etc. Biomonitoring of metal in blood and urine also has certain limitations. Studies have shown that metal content in urine fluctuates greatly on a daily and weekly basis, and the results lack reliability [18]. Recently, a new approach of metabonomics has been proposed to evaluate the biological effects of occupational exposure. Metabonomics uses omics measurements to obtain small molecules present in biological samples, known as metabolites, which provide an internal indicator of molecular change. Thus, the potential mode of action and the pathway of change of metals in organisms can be identified, which can be used to identify the biological effects of exposure to toxins. This enhanced the stability of the monitoring of internally exposed metals, to a certain extent [19].

In this study, we evaluated the environmental exposure of workers to metals in a factory through a case-control study. Then the relationship between single metal exposure and hypertension was assessed. Finally, the effects of exposure to four metals on hypertension prevalence and blood pressure levels were estimated. This study attempts to explore the potential association between metal exposure in the working environment and the risk of hypertension.

## 2. Materials and Methods

### 2.1. Study Population

We chose workers in a factory in Wuhan for the study. Each participant completed a questionnaire that included detailed information on age, education, smoking, and drinking status. Height, weight, and blood pressure were determined by trained professionals. BMI was calculated according to height and weight, and was classified as lean, normal, or overweight (<18.5, 18.5–24, >24 kg/m^2^). Smoking and drinking were divided into never, current, and past. We conducted the research on manufacturing shop and maintenance shop workers. The collected questionnaires were screened and the missing values were removed; 138 patients with hypertension were selected as the case group, and then the remaining non-hypertensive subjects were matched 1:1 by propensity matching to 138 as the control group; data on 276 workers were withheld for further analysis.

The propensity score matching process was completed using the “Propensity Score Matching” option in SPSS software. Gender and age were used as matching variables (predictors), and match tolerance was set to 0.02 to achieve 1:1 matching between the case group and the control group in the matching process.

### 2.2. Determination of Blood Pressure

Hypertension was defined as a systolic blood pressure of 140 or greater or a diastolic blood pressure of 90 or greater, as well as a doctor’s diagnosis of hypertension or the use of medication for hypertension [20]. Blood pressure was measured by a professional. After a 5-min rest, an appropriate size cuff was placed on the exposed right arm of the participant, and blood pressure was measured three times in a sitting position, with systolic and diastolic blood pressure as the average of the measurements.

### 2.3. Metal Concentration in the Air

The sampling points were set up according to the specifications (GBZ 159−2004) of air sampling for hazardous substances monitoring in the workplace. Personal sampling was conducted at the height of the personal breathing zone; airborne dust and metals were collected for at least 120 min using polyvinyl chloride and a cellulose acetate filter at 1 L/min using Gilair Plus. Dust mass was measured with a ppm balance and metal content was detected using an inductively coupled plasma emission spectrometer (Perkineimer Avio200). Metal exposure was classified into five levels based on occupational exposure to hazardous chemical elements: Level 0 [≤1% occupational exposure limit (OEL)] indicated almost no contact; Level 1 (>1%, ≤10% OEL) indicated extremely low exposure, no correlation; grade 2 (>10%, ≤50% OEL) indicated no apparent health impact, grade 3 (>50%, ≤OEL) indicated significant exposure requiring action to limit activity, level 4 (>OEL) indicated a greater health hazard after exposure [21].

### 2.4. Statistical Analysis

Descriptive statistics were performed based on the demographic characteristics of the participants. Differences in continuous or categorical variables between hypertensive and non-hypertensive groups were compared using parametric or nonparametric methods. The association between metal exposure grade and the risk of hypertension was assessed by calculating odds ratios (ORs) using conditional logistic regression models. The models included age, BMI, smoking status, alcohol consumption, Cr, and Ni. All statistical analyses were determined using SPSS. All statistical analyses were two-sided tests, and *p*-values < 0.05 were considered significant.

## 3. Results

### 3.1. Characteristics of the Study Population

The basic demographic characteristics of the study population are shown in Table 1. A total of 276 participants were included, 138 hypertensives and 138 non-hypertensives. The mean age in the hypertensive workers was 46.7 ± 7.31 years, while the mean age was 46.4 ± 7.84 years in the control group. There were no significant differences between the two groups in education level, smoking, drinking, or physical activity. Compared with the control group, the BMI of hypertensive patients may be higher (*p* < 0.001).

### 3.2. Heavy Metal Exposure Levels and the Risk of Hypertension

We divided workers into four categories: non-welder and welder in a manufacturing workshop, non-welder and welder in a repair workshop, and then monitored the concentrations of the heavy metal ions Cr, Fe, Mn, and Ni in their working environments (Table 2). Welders in the manufacturing workshop and repair workshop were exposed to Cr ion concentrations of 0.014135 mg/m^3^ and 0.019987 mg/m^3^, respectively, both of which were level 3 exposures. The exposure concentration of Fe is relatively high. Except for the non-welders in the manufacturing workshop, all workers in the workshop had reached level 4 exposure. Welders also had level 4 Mn exposure, while non-welders in the manufacturing shop and maintenance shop had level 1 and level 3 exposure, respectively. The Ni concentration in the work shop was detected to be low. Except for welders in the maintenance workshop and the welders in the repair workshop, whose exposure levels reached grade 1, the rest of the workers were all at level 0 exposure risk.

In the above table, workers were stratified according to the risk levels of heavy metal exposure in different workshops and types of work, and the relationship between the risk levels of heavy metal exposure in the working environment and hypertension was analyzed (Table 3). The results showed that the risk levels of exposure to Cr, Fe, Mn, and Ni ions were different from the risk of hypertension (*p* < 0.001). This suggested that the heavy metals Cr, Fe, Mn, and Ni ions may be associated with the risk of hypertension.

### 3.3. Multivariate Analysis and Hypertension

We further conducted multivariate conditional logistic regression analysis, including age, BMI, smoking, and drinking as confounding factors, to explore the relationship between environmental heavy metal exposure and the risk of hypertension (Table 4). In multivariate analysis, BMI was still positively associated with increased risk of hypertension compared with univariate analysis, and the higher the BMI, the higher the risk of hypertension (OR = 1.06, *p* = 0.005). In the models, the higher the level of Cr exposure of the four heavy metals studied, the higher the risk of hypertension. The risk of hypertension was 1.85 times higher in level 3 than in level 1 (*p* = 0.006). In the regression analysis, there was multicollinearity between Fe and Mn in the model and Cr, so Fe and Mn were not included in the model. We then included Mn and Fe in the analyses, respectively, and the results showed that Mn had the same effect on hypertension as Cr, with the highest exposure levels increasing the risk of hypertension. Inconsistencies with the results of the univariate analysis, the level of Fe and Ni ion exposure was not significantly associated with the risk of hypertension (*p* > 0.05).

## 4. Discussion

In this study, we examined the relationship between four essential metals and hypertension. We found that the incidence of hypertension may increase with increasing levels of Cr exposure. Consistent with the conclusion of this study, long-term residence in areas exposed to Cr has a higher risk of hypertension [22]. Cr is a transition metal, and its toxicity is related to its valence state. Trivalent Cr is beneficial to the human body, while hexavalent Cr is easily absorbed and accumulated, and its toxicity is 100 times that of trivalent Cr [23]. Some researchers treated mice with Cr and found reduced expression of RKIP (which affects the signaling and contractile activity of heart muscle cells) in the heart (in vivo) and in cardiomyocytes (in vitro), Cr may cause a decline in cardiovascular function [24]. High doses of trivalent Cr supplements can reduce blood pressure in rats [25]. A large sample study in rural China indicated that Cr concentration may be independently associated with hypertension, inconsistent with the results of our study; patients with hypertension were exposed to lower concentrations of Cr than controls [7]. Cr is often considered a cause of cardiovascular disease due to its association with the regulation of lipid and glucose metabolism, while Robert Amadu Ngala et al. [15] showed that high plasma Cr appeared to reduce the risk of hypertension, and low Cr levels were associated with increased systolic and diastolic blood pressure. In a baseline study, Cr was significantly associated with hypertension in a univariate model; however, Cr was not significantly associated with hypertension risk when all metals were included in the same model [26].

Our study suggested an association between Mn exposure and hypertension, while no effect of Fe or Ni and hypertension was found. Fe mainly exists in the form of hemoglobin and ferritin in the body, which is involved in the transport and storage of oxygen and is one of the essential elements of the human body [27]. Mn is a component of many enzymes and immune system compounds, but long-term exposure to Mn can cause neurological symptoms [28]. A review of manganese exposure has shown that manganese is able to rapidly accumulate in heart tissue and cause acute or subacute cardiovascular disease. These toxic results appear to be related to mitochondrial damage caused by manganese and mutual effect with calcium channels in the cardiovascular system [29]. The result of this study was that Mn could play a role in elevating blood pressure in humans, but the results of previous studies might be inconsistent. Urine Mn could control blood pressure and prevent hypertension, and levels were inversely associated with cardiovascular disease risk in a national study of older adults in the United States [14]. In addition, Mn concentration in the hair of hypertensive patients was significantly lower than that of normal controls [30]. In a Gulf long-term follow-up study, researchers found that Mn might be positively associated with the risk of hypertension, especially after racial stratification, with a stronger association between blood Mn and hypertension in Black participants than in Whites [22]. Fe accumulation in the body could lead to inflammation and endothelial dysfunction, which lead to increased blood pressure and the incidence of hypertension [9,10]. Cr, Fe, Mn, and Ni exposure levels were all associated with hypertension risk according to the results of the single-metal model, whereas no association between Ni and hypertension was observed in the multivariate model. There was no significant difference in Ni exposure between hypertensive and normotensive subjects, consistent with the findings of Gonzalez Munoz et al. [30]. Polymetallic models can be helpful in determining the adjusted effects of multiple metals, but this association may affect the final results when variables are not independent risk factors for related outcomes. Consistent with the results of many previous studies, BMI was linearly related to blood pressure, and obesity could promote the increase of blood pressure [2]. The same conclusion was also reached in this study, and obesity was significantly associated with the development of hypertension.

Although it was reported that essential metals were a key factor in the normalization of blood pressure, increasing evidence showed that both high and low levels of essential metals were detrimental to human health [31]. A recent epidemiology study reported a positive association between metal mixtures in the blood and blood pressure, with blood lead considered the most important factor compared to other metals [32]. Base metals such as Cr, Mn, Fe, zinc, and copper were key factors influencing blood pressure levels, and when these elements were insufficient or too high, they might cause adverse effects and damage the cardiovascular system [26]. The underlying mechanism of the association between essential metals and hypertension is not fully understood, and many researchers suggested that it might be related to the antioxidant effects of Cr, Mn, and Fe. Cr reduces sensitivity to cardiovascular disease by inhibiting protein glycosylation and oxidative stress. High Mn intake could inhibit the development of hypertension by increasing the activity of Mn superoxide dismutase (MN-SOD), reducing O_2_ and promoting the activity of NO [7]. Fe plays a key role in the induction of oxidative stress. Accumulation of these metals in the body might increase blood pressure levels by inducing endothelial dysfunction and inflammatory activity [26].

The number of participants in our study was not large enough, and the participants were occupational groups that had more opportunities to contact metals than the general population. Therefore, the results of this study had certain limitations when applied to the general population. We used the average exposure level of a workshop worker as the metal content in the air, not the individual exposure level of the worker; individual exposure to metal concentration varied according to the worker’s activity and behavior, so there would be some differences. Cr metal due to different valence and toxicity differences, detection of hexavalent Cr will inevitably have trivalent Cr mixed, so there was a certain impact on the results. In addition, we only considered metals in the work environment, but metal exposure does not only come from this source, but also from diet, soil, etc. This study included data on environmental exposures only, and did not measure metals in plasma or urine, so the results should be interpreted with caution. This was a case-control study and did not consider the temporal relationship between metal exposure and hypertension, so no causal relationship can be established and further prospective study designs are needed to confirm our findings.

## Figures and Tables

**Table 1:** General characteristics among the study population (n = 276).

Characteristic	Non-Hypertensive (n = 138)	Hypertensive (n = 138)	Total	*p*-Value
Age (years)	46.4 ± 7.84	46.7 ± 7.31	46.6 ± 7.57	0.703
Education (*n*, %)				0.727
Primary school or lower	2 (1.4)	0	2 (0.7)	
Middle school	33 (23.9)	32 (23.2)	65 (23.6)	
High school	71 (51.4)	74 (53.6)	145 (52.5)	
Junior college or higher	32 (23.2)	32 (23.2)	64 (23.2)	
BMI (kg/m^2^)	24.6 ± 3.30	26.5 ± 4.00	25.6 ± 3.78	<0.001
BMI grouping (*n*, %)				0.003
<18.5 kg/m^2^	7 (5.1)	0	7 (2.5)	
18.5–25 kg/m^2^	66(47.8)	55(39.9)	121 (43.8)	
>25 kg/m^2^	65 (47.1)	83 (60.1)	148 (53.6)	
Smoking status (*n*, %)				0.765
Never	43 (31.2)	45 (32.6)	88 (31.9)	
Current	91 (65.9)	87 (63)	178 (64.5)	
Past	4 (2.9)	6 (4.3)	10 (3.6)	
Alcoholic intake status (*n*, %)				0.141
Never	92 (66.7)	76 (55.1)	168 (60.9)	
Current	44 (31.9)	59 (42.8)	103 (37.3)	
Past	2 (1.4)	3 (2.2)	5 (1.8)	
Exercise (*n*, %)				0.66
No	110 (79.7)	107 (77.5)	217 (78.6)	
Yes	28 (20.3)	31 (22.5)	59 (21.4)	

The *t*-test was used for differences in age and BMI, the chi-square test was used for differences in smoking status and exercise, and Fisher’s exact test was used for education level, BMI grouping, and alcohol intake status.

**Table 2:** Exposure levels to heavy metals in the work environment (mg/m^3^).

	Mean	Min	Max	PC-TWA (8 h/40 h)	PC-TWA (72 h)	PC-TWA (91 h)	Risk Level
Cr							
Non-welder in manufacturing workshop	0.000603	0.00031	0.001075	0.05	0.02085	0.0132	1
Welder in manufacturing workshop	0.014135	0.002283	0.021583	0.05	0.02085	0.0132	3
Non-welder in repair workshop	0.007699	0.005342	0.010055	0.05	0.02085	0.0132	2
Welder in repair workshop	0.019987	0.00578	0.034193	0.05	0.02085	0.0132	3
Fe							
Non-welder in manufacturing workshop	0.091064	0.057313	0.154414	0.25	0.10425	0.066	2
Welder in manufacturing workshop	2.158942	0.443535	3.18455	0.25	0.10425	0.066	4
Non-welder in repair workshop	1.262596	0.871609	1.653583	0.25	0.10425	0.066	4
Welder in repair workshop	3.792913	1.305619	1.305619	0.25	0.10425	0.066	4
Mn							
Non-welder in manufacturing workshop	0.010721	0.004992	0.019362	0.15	0.06255	0.0396	1
Welder in manufacturing workshop	0.282111	0.039982	0.453504	0.15	0.06255	0.0396	4
Non-welder in repair workshop	0.124932	0.094887	0.154976	0.15	0.06255	0.0396	3
Welder in repair workshop	0.34695	0.092056	0.601845	0.15	0.06255	0.0396	4
Ni							
Non-welder in manufacturing workshop	0.000299	0.00017	0.000542	1	0.417	0.264	0
Welder inmanufacturing workshop	0.006827	0.002015	0.009531	1	0.417	0.264	0
Non-welder in repair workshop	0.004897	0.004321	0.005474	1	0.417	0.264	0
Welder in repair workshop	0.011883	0.007891	0.015875	1	0.417	0.264	1

**Table 3:** Exposure risk level and hypertension.

Risk Level	Non-Hypertensive(n = 138)	Hypertensive(n = 138)	Total	*p*-Value
Cr (*n*, %)				<0.001
1	80 (58)	50 (36.2)	130 (47.1)	
2	42 (30.4)	34 (24.6)	76 (27.5)	
3	16 (11.6)	54 (39.1)	70 (25.4)	
Fe (*n*, %)				<0.001
2	80 (58)	50 (36.2)	130 (47.1)	
4	58 (42)	88 (63.8)	146 (52.9)	
Mn (*n*, %)				<0.001
1	80 (58)	50 (36.2)	130 (47.1)	
3	42 (30.4)	34 (24.6)	76 (27.5)	
4	16 (11.6)	54 (39.1)	70 (25.4)	
Ni (*n*, %)				<0.001
0	135 (97.8)	119 (86.2)	254 (92)	
1	3 (2.2)	19 (13.8)	22 (8)	

The *p* values in this table were obtained by the chi-square test.

**Table 4:** Multivariate logistic regression and hypertension.

Variables	OR (95% CI)	*p*-Value
Age	1.01 (0.99, 1.04)	0.350
BMI	1.06 (1.02, 1.11)	0.005
Smoking		0.886
Smoking(1)	0.96 (0.67, 1.39)	0.839
Smoking(2)	1.19 (0.48, 2.94)	0.701
Drinking		0.439
Drinking(1)	1.26 (0.88, 1.80)	0.201
Drinking(2)	1.05 (0.30, 3.61)	0.942
Cr		0.019
Cr(1)	1.15 (0.74, 1.79)	0.527
Cr(2)	1.85 (1.20, 2.86)	0.006
Ni	1.20 (0.67, 2.14)	0.550

Conditional logistics regression was used to obtain *p* values. Due to the multicollinearity between iron and manganese and chromium, only chromium was included in the model.

## Data Availability

The original data presented in this study will be provided by the authors.
